# Supplementary figures and images for: The mitochondrial HSP90 paralog TRAP1 forms an OXPHOS-regulated tetramer and is involved in mitochondrial metabolic homeostasis
Source: BMC Biol. 2020 Jan 27;18:10. doi: 10.1186/s12915-020-0740-7 (PMC6986101; doi:10.1186/s12915-020-0740-7)

Figure S1

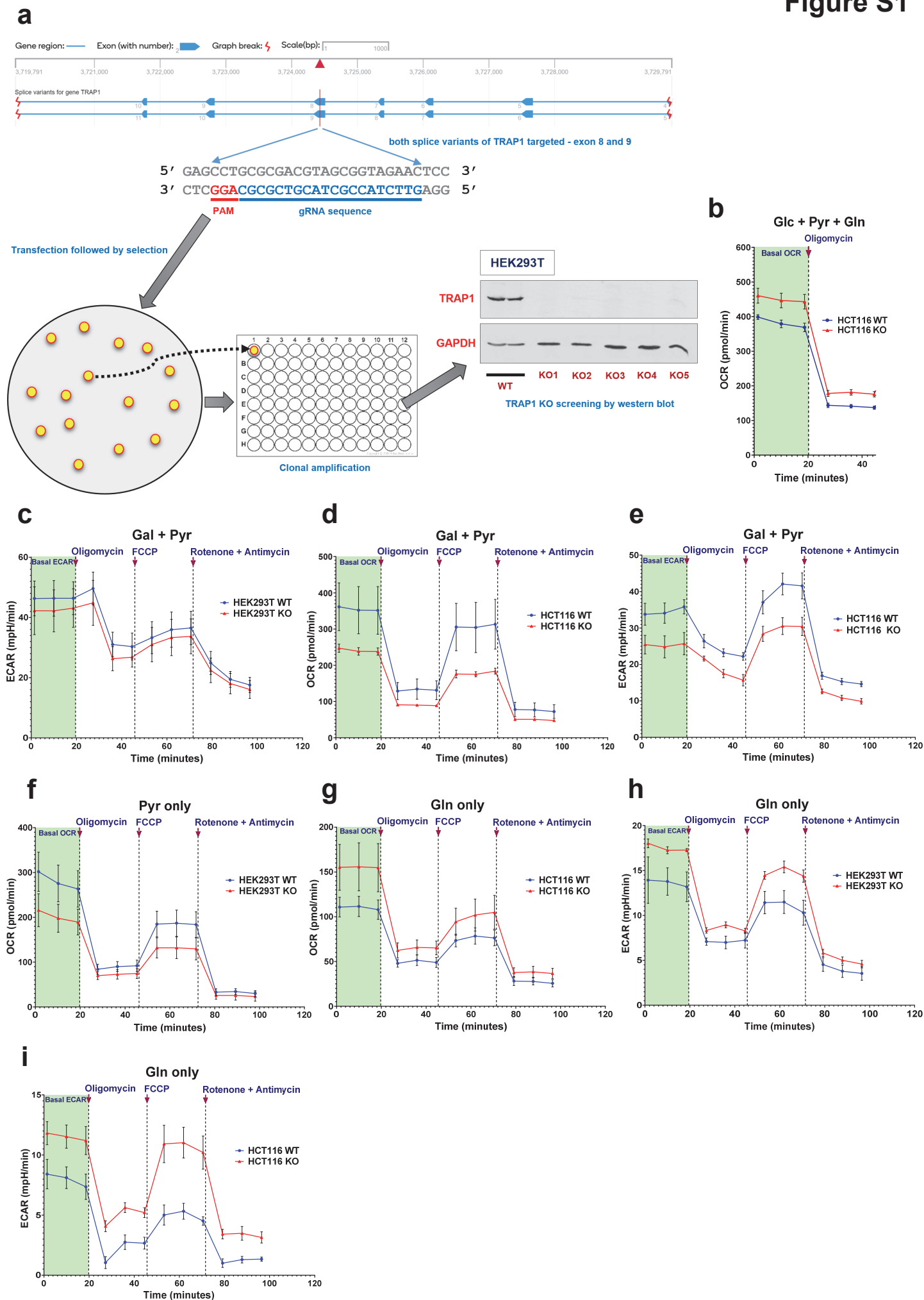

Supplement: Supplementary file 1 — Additional file 1: Figure S1. Generation of TRAP1 KO cells and additional metabolic profiling. (a) Workflow for the generation of CRISPR/Cas9-mediated TRAP1 KO clones. Unlike HEK293T and HCT116 clones, A549 and UMUC3 TRAP1 KO clones were isolated by fluorescence-activated cell sorting using a vector allowing mCherry expression (see Additional file 16: Table S9). (b) OCR traces of WT and KO HCT116 cells with Glc + Pyr + Gln as carbon sources. (c-i) OCR and ECAR traces of WT and KO HEK293T or HCT116 cells with different primary carbon sources. [file 12915_2020_740_MOESM1_ESM.pdf]

Carbon flow from <sup>13</sup>C Gln into the TCA cycle

Figure S2

a

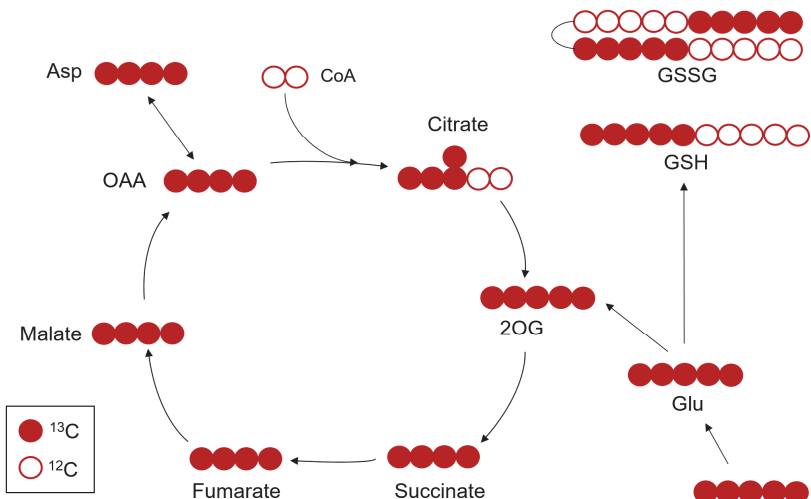

b

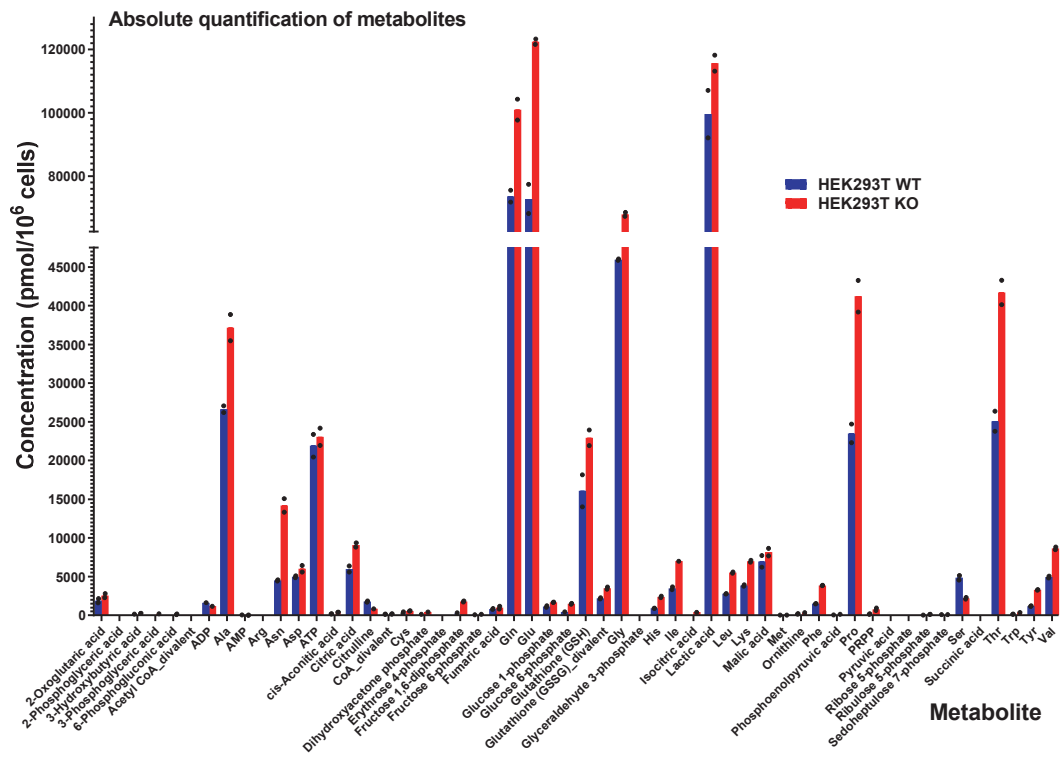

c

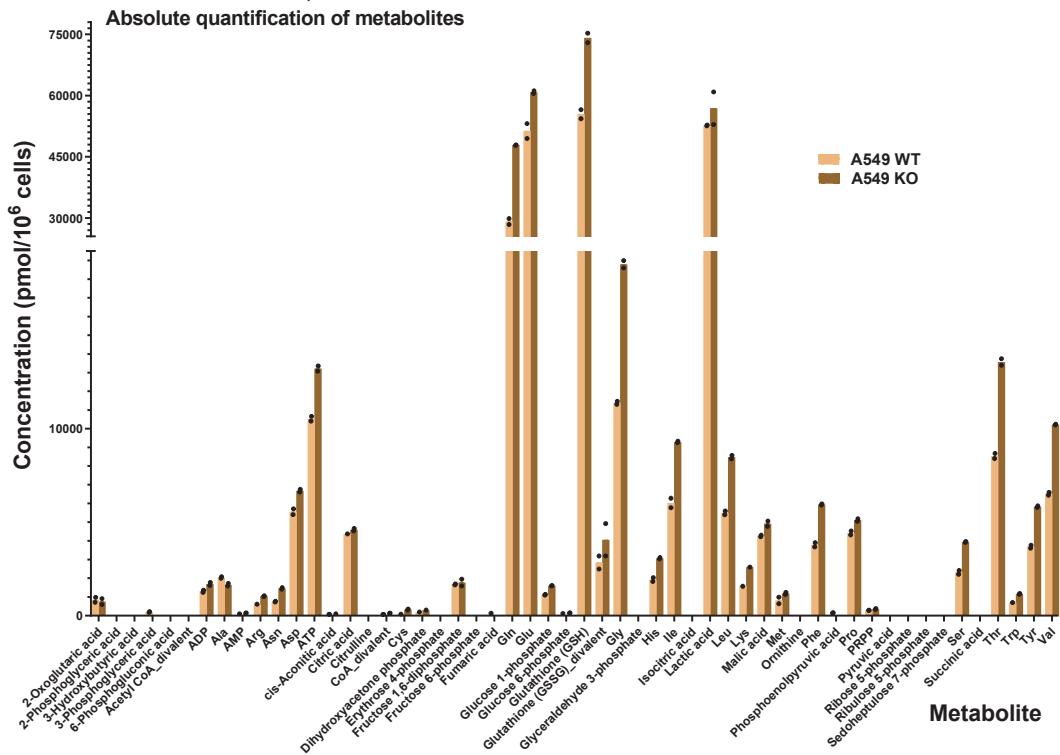

Supplement: Supplementary file 2 — Additional file 2: Figure S2. Carbon flux and total quantitation of target metabolites. (a) Schematic metabolic map showing the flow and distribution of 13C atoms in metabolites of the TCA cycle when cells consume 13C-Gln. Note that most of these metabolites traced with 13C-Gln were found to be upregulated in TRAP1 KO cells. (b, c) Total quantitation of target metabolites in WT and KO HEK293T and A549 cells. Note that this is “total” quantitation and should not be confused with 13C tracing. Total quantitation must be combined with the information provided in Additional file 4: Table S2 to infer metabolites with increased 13C incorporation. Data points on bar graphs indicate metabolite concentration per 106 cells from each biological replicate (n = 2). [file 12915_2020_740_MOESM2_ESM.pdf]

**Figure S3**

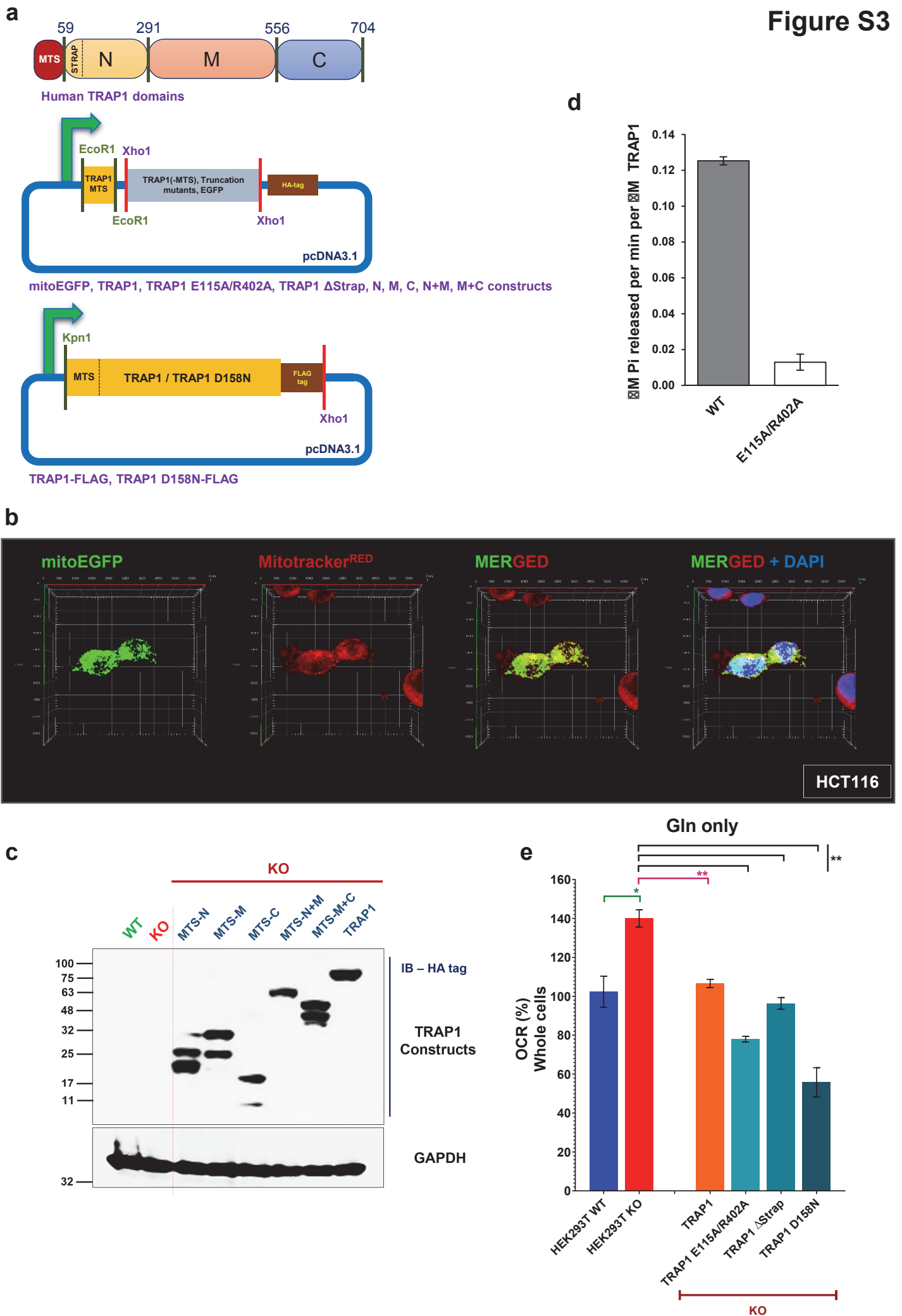

Supplement: Supplementary file 5 — Additional file 5: Figure S3. TRAP1 truncation and point mutants. (a) Schematic representation of the constructs for expression of mitochondrially targeted TRAP1 and EGFP. (b) Fluorescence micrographs showing proper targeting of mitoEGFP to mitochondria. Mitochondria are revealed with MitotrackerRED. (c) Expression analysis of TRAP1 truncation mutants by immunoblotting with an antibody to their HA-tag. (d) ATPase activity assay for the TRAP1 double mutant E115A/R402A. (e) Quantitation of basal respiration rates in WT versus KO HEK293T cells expressing the indicated proteins. Note that all ATPase mutants can rescue the KO phenotype to WT levels. [file 12915_2020_740_MOESM5_ESM.pdf]

Figure S4

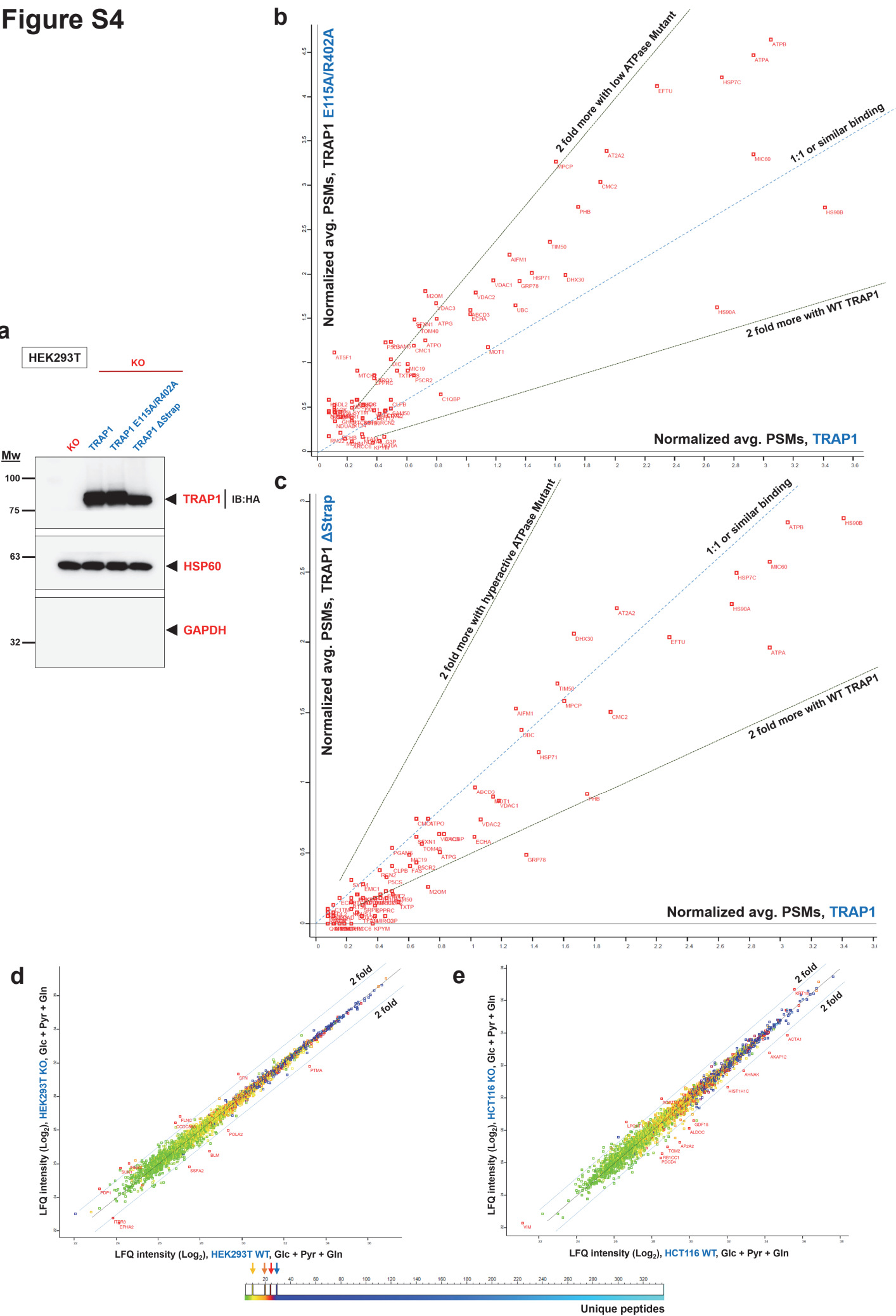

Supplement: Supplementary file 6 — Additional file 6: Figure S4. Analysis of the whole cell proteome and TRAP1-associated proteins. (a) Control immunoblot performed to check TRAP1 WT and mutant expression in the KO cells used for the IP-MS experiments. (b, c) Comparative relative abundance of proteins immunoprecipitated with the indicated TRAP1 ATPase muatnts or WT TRAP1. The scatterplot was generated as mentioned in the legend to Fig. 4a. (d, e) Scatter plots comparing the levels (LFQ intensities) of the 3679 high confidence proteins between WT and KO HEK293T or HCT116 cells. Note that proteins highlighted in red above or below the 2-fold cutoff did not change consistently between the two cell lines. [file 12915_2020_740_MOESM6_ESM.pdf]

Figure S5

a

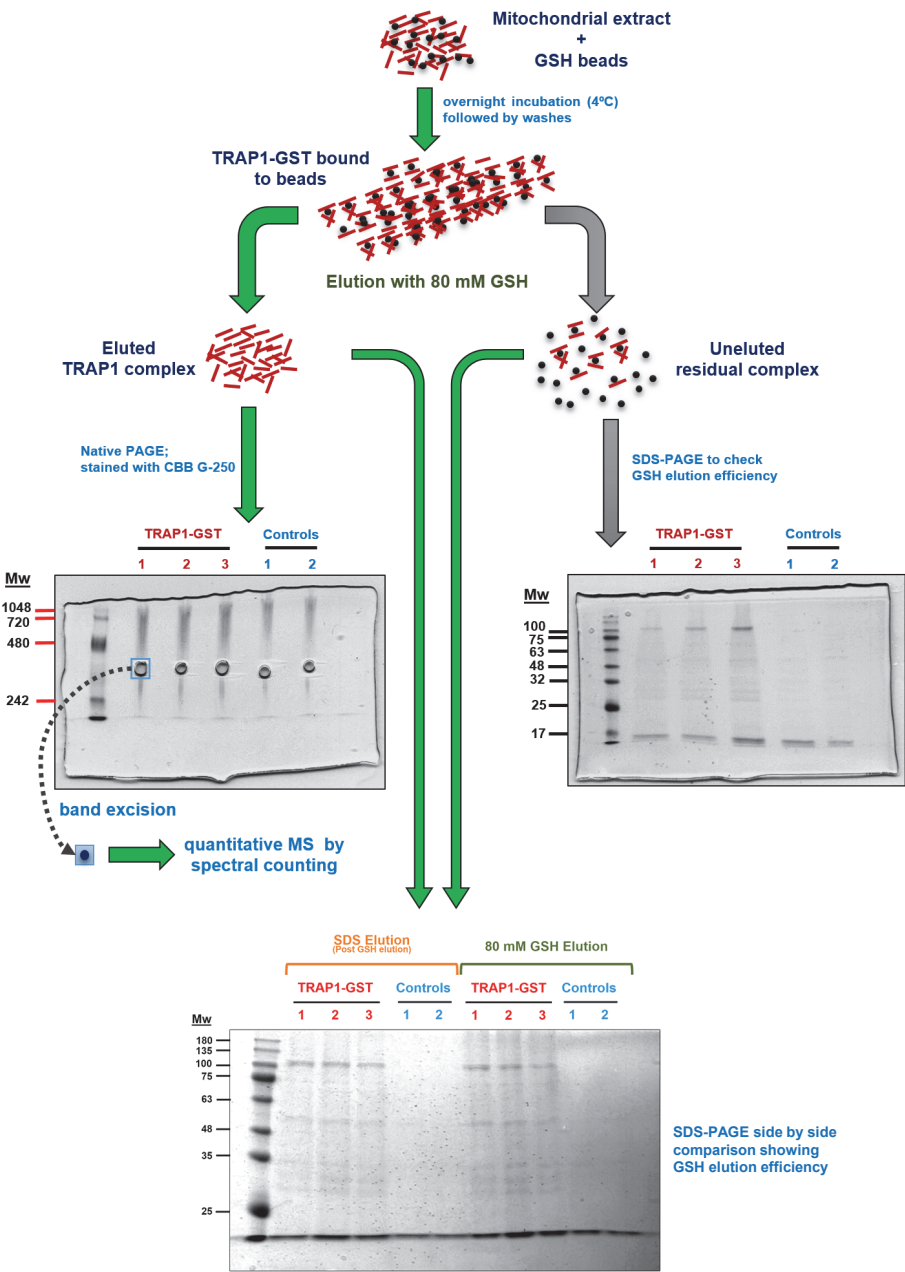

b

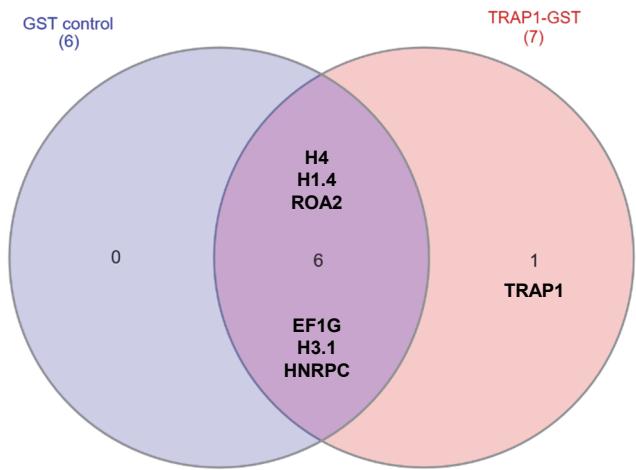

c

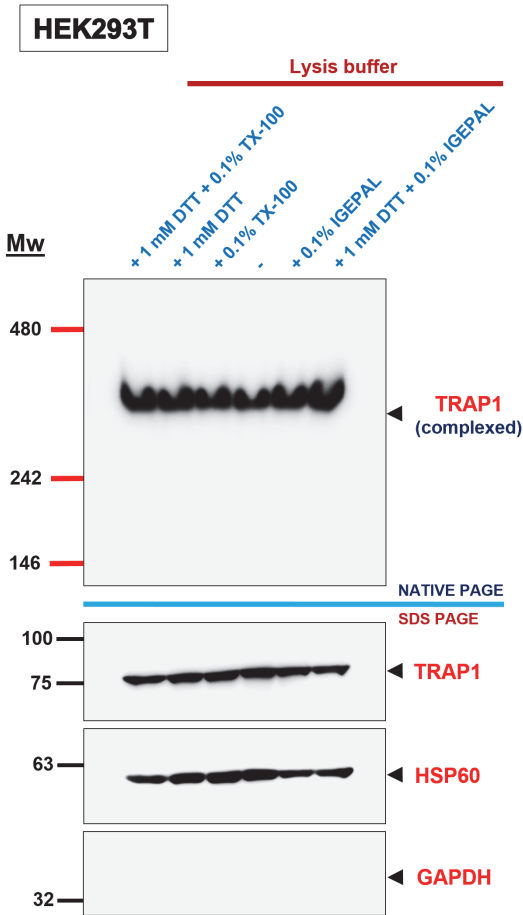

Supplement: Supplementary file 12 — Additional file 12: Figure S5. An extension of Figure 5 showing TRAP1-GST pulldown MS strategy and analysis, and a control experiment for mitochondrial lysis conditions. (a) TRAP1-GST pulldown strategy. (b) Venn diagram of the proteins identified by the MS analysis. Note that TRAP1 peptides are the only unique ones in the TRAP1-GST pulldown samples compared to the GST controls. (c) TRAP1 complexes from mitochondria, lysed with the indicated buffers, analysed by native PAGE and SDS-PAGE. The standard lysis buffer contained 1 mM DTT and 0.1% Triton X-100 (first lane); variations as indicated. IGEPAL, IGEPAL CA-630 (Sigma-Aldrich #I30211). [file 12915_2020_740_MOESM12_ESM.pdf]

# Figure S6

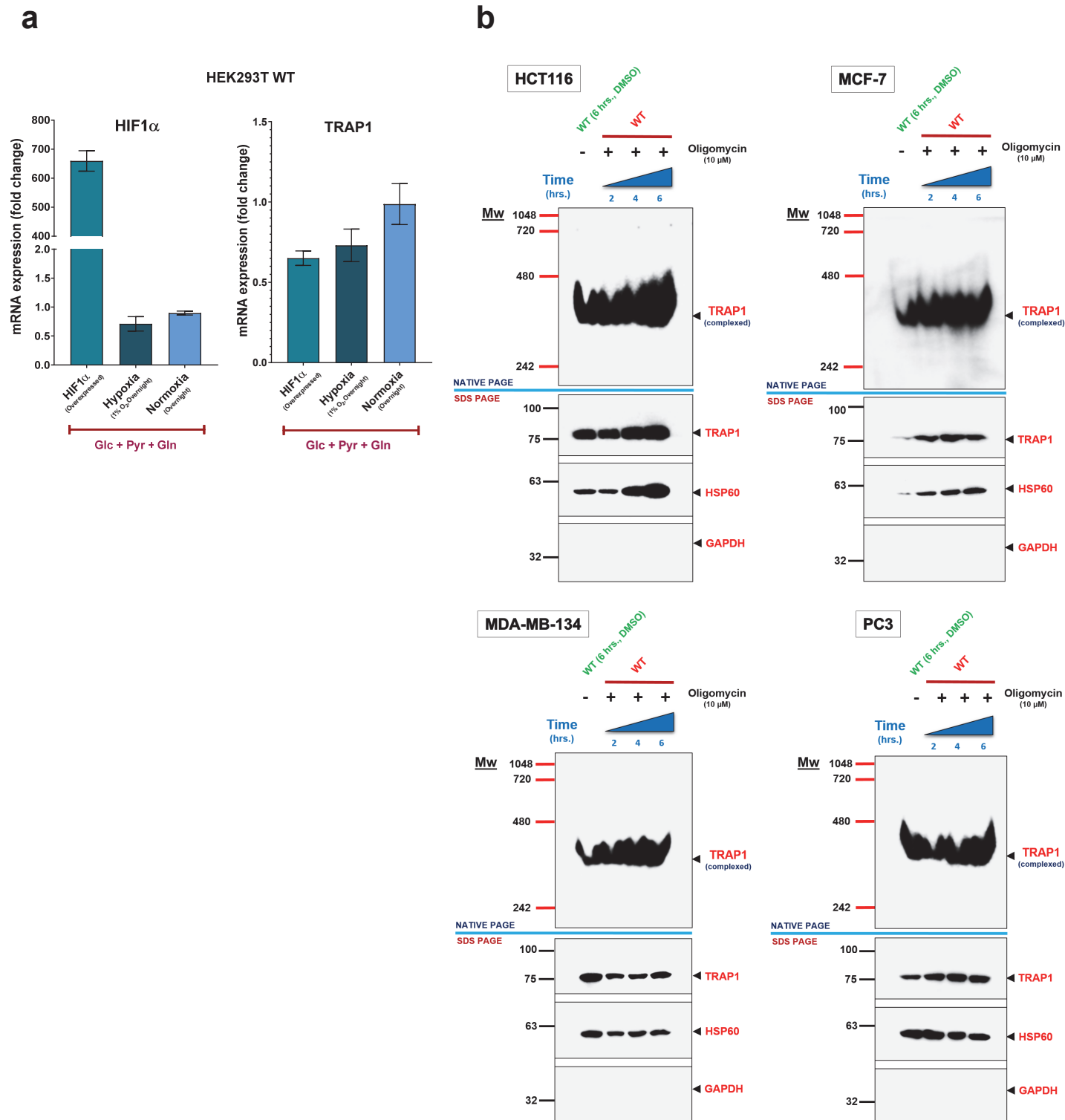

Supplement: Supplementary file 14 — Additional file 14: Figure S6. TRAP1 is not induced by HIF1α and the TRAP1 complex is ubiquitous. (a) Quantitative RT-PCR analysis of the mRNA levels for HIF1α and TRAP1. All data are reported as means ± SEM (n = 3). (b) Analysis of TRAP1 complexes from indicated cell lines by native PAGE and SDS-PAGE. [file 12915_2020_740_MOESM14_ESM.pdf]
