# Supplementary material for: The mitochondrial HSP90 paralog TRAP1 forms an OXPHOS-regulated tetramer and is involved in mitochondrial metabolic homeostasis
Source: BMC Biol. 2020 Jan 27;18:10. doi: 10.1186/s12915-020-0740-7 (PMC6986101; doi:10.1186/s12915-020-0740-7)
Supplement: Supplementary file 15 — Additional file 15: Figure S7. Differential effects of drug treatments on the TRAP1 complex. [file 12915_2020_740_MOESM15_ESM.pdf]

Figure S7

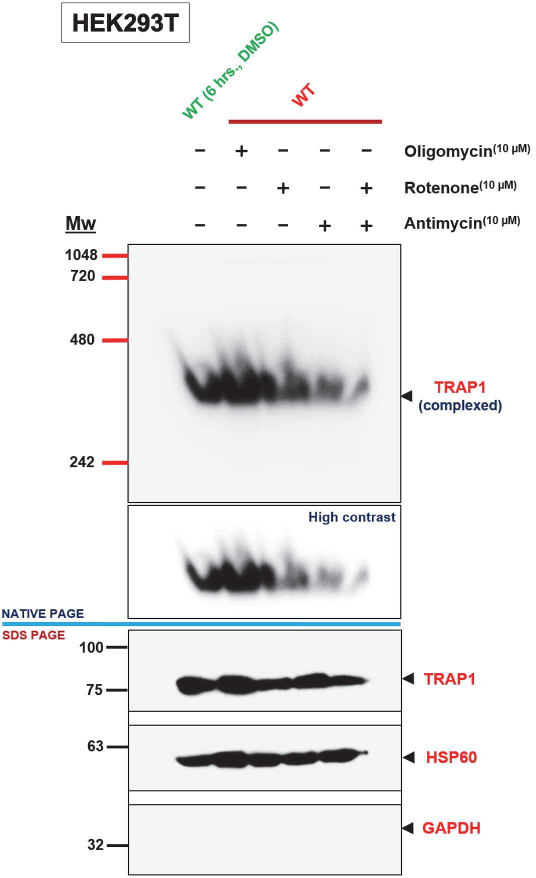
HEK293T
WT (6 hrs., DMSO)
WT
− + − − − Oligomycin(10 μM)
− − + − + Rotenone(10 μM)
− − − + + Antimycin(10 μM)
Mw
1048
720
480
242
TRAP1 (complexed)
High contrast
NATIVE PAGE
SDS PAGE
100
75
TRAP1
63
HSP60
32
GAPDH
